# Colonization of *Phlebotomus papatasi *changes the effect of pre-immunization with saliva from lack of protection towards protection against experimental challenge with *Leishmania major *and saliva

**DOI:** 10.1186/1756-3305-4-126

**Published:** 2011-07-04

**Authors:** Sami Ben Hadj Ahmed, Belhassen Kaabi, Ifhem Chelbi, Saifeddine Cherni, Mohamed Derbali, Dhafer Laouini, Elyes Zhioua

**Affiliations:** 1 Laboratory of Vector Ecology, Institut Pasteur de Tunis, Tunis, Tunisia; 2 Department of Biology, University of Gafsa, Gafsa, Tunisia; 3 Laboratory of Epidemiology and Ecology of Parasites, Institut Pasteur de Tunis, Tunis, Tunisia; 4 Laboratory of Immuno-Pathology, Vaccinology, and Molecular Genetics, Institut Pasteur de Tunis, Tunis, Tunisia

## Abstract

**Background:**

Sand fly saliva has been postulated as a potential vaccine or as a vaccine component within multi component vaccine against leishmaniasis. It is important to note that these studies were performed using long-term colonized *Phlebotomus papatasi*. The effect of sand flies colonization on the outcome of *Leishmania *infection is reported.

**Results:**

While pre-immunization of mice with salivary gland homogenate (SGH) of long-term colonized (F5 and beyond) female *Phlebotomus papatasi *induced protection against *Leishmania major *co-inoculated with the same type of SGH, pre-immunization of mice with SGH of recently colonized (F2 and F3) female *P. papatasi *did not confer protection against *L. major *co-inoculated with the same type of SGH. Our data showed for the first time that a shift from lack of protection to protection occurs at the fourth generation (F4) during the colonization process of *P. papatasi*.

**Conclusion:**

For the development of a sand fly saliva-based vaccine, inferences based on long-term colonized populations of sand flies should be treated with caution as colonization of *P. papatasi *appears to modulate the outcome of *L. major *infection from lack of protection to protection.

## Background

Leishmaniasis is a neglected tropical disease affecting two million people per year worldwide [1]. Sand flies are the main vector of *Leishmania*, the etiologic agent of leishmaniasis. Depending on the sand fly and *Leishmania *species, different clinical forms of the disease from cutaneous, muco-cutaneous, and visceral occur. Control of leishmaniasis is based largely on chemical therapy and vector control measures. However, these methods have met with variable success [2,3]. To date no effective vaccine is available [4].

During blood meals, sand flies salivate into the host's skin. Beyond the functions associated with overcoming vertebrate homeostasis, sand fly saliva modulates the inflammatory response of the host and displays many immunomodulatory properties [5]. Sand fly saliva contains an array of bioactive molecules that allow the vector to successfully obtain a blood meal and enhance transmission of *Leishmania *promastigotes into a vertebrate host [5]. Among some of the most abundant molecules are anticlotting, antiplatelet, and vasodilatory compounds that increase the hemorrhagic pool where sand flies feed [5].

Sand fly saliva was shown to exacerbate *Leishmania *infection [6,7]. Several studies reported that pre-immunization with salivary gland homogenates (SGH), salivary component, or pre-exposition to uninfected bites of *Phlebotomus papatasi *provided significant protection against infection with *Leishmania major*, the etiologic agent of zoonotic cutaneous leishmaniasis (ZCL) [8-10]. All these studies were performed with long-term colonized female *P. papatasi*. Recent data by our group demonstrated that pre-immunization with SGH of wild-caught or with SGH of recently colonized (F1) female *P. papatasi *did not confer protection against *L. major *compared to a significant protection obtained with SGH of long-term colonized ones (F29) [11]. Therefore, during the colonization process, the effect of SGH shifts from lack of protection towards protection. As most studies conducted on sand fly biology rely on colonized sand flies, our principal objective was to determine at which generation this shift from lack of protection to protection against lesion development and parasite load occurs.

## Materials and methods

### Sand flies, parasites and animals

Wild sand flies were collected using CDC light traps from an animal shelter located in the village of Felta (governorate of Sidi Bouzid), a highly endemic focus of ZCL [12]. *Phlebotomus papatasi *was found to be the most abundant sand fly species caught in this area [12]. A new colony of *P. papatasi *(Tunisian strain) was initiated at the Vector Ecology Laboratory of the Institut Pasteur de Tunis [13]. Six generations of *P. papatasi *(F2, F3, F4, F5, F6 and F14) were used in this study. Since the number of protein components in SGH of *P. papatasi *increases with age and produces a typical electrophoretic pattern within three to five days after emergence [14], sand flies were dissected at three to seven days after emergence. Salivary glands were removed under a stereo microscope in cold phosphate-buffer saline (PBS) (8 mM Na_2_HPO_4_, 1.75 mM KH_2_PO_4_, 0.25 mM KCl, 137 mM), (pH 7.4), and stored in groups of 20 pairs in 20 μl of PBS (pH 7.4) at -70°C. Immediately before use, 20 pairs of salivary glands were disrupted in 100 μl of PBS buffer by three cycles of freezing-thawing.

A highly virulent strain of *L. major *MHOM/TN/95/GLC94, isolated from a Tunisian patient was used in this study [15]. Amastigotes were obtained after passage in BALB/c mice in the footpad and harvested from skin lesions by differential centrifugation. Promastigotes were grown on NNN medium at 26°C and then progressively adapted to RPMI 1640 medium (Sigma, St Louis, Mo.) containing 2 mM L-glutamine, 100 U of penicillin/ml, 100 μg of streptomycin/ml, and 10% heat-inactivated foetal calf serum (complete medium). Promastigotes were collected while in the stationary growth phase (enriched metacyclic) by centrifugation (3000 × g, 10 min, 14°C), washed three times in PBS and re-suspended to the appropriate concentration.

BALB/c mice were bred in the animal facility the Institut Pasteur de Tunis under pathogen-free conditions. Female mice aged between six and eight weeks were used in this study. All experiments involving BALB/c mice were performed in accordance with protocols approved by the Institutional Animal Care and Use Committee of Pasteur Institute of Tunis.

### Immunizations with SGH and challenge

Mice were anaesthetized by subcutaneous injection of 200 μl of ketamine (10 mg/ml) (Merial, Lyon, France), and immunized intradermally in the right ear with the equivalent of two pairs of salivary glands in 10 μl of PBS. Six groups of 10 mice each were pre-immunized with SGH obtained from sand fly generations F2, F3, F4, F5, F6, and F14, once a week for two weeks. In the fourth week, the groups were challenged with 10^6 ^*L. major *promastigotes co-inoculated with the same type of the SGH used in pre-immunizations. Six control groups of 10 mice each were injected with PBS instead of SGH and challenged with promastigotes co-inoculated with each of the six types of SGH. Female BALB/c mice pre-immunized with SGH and six control groups of mice were challenged with a mixture of two pairs of salivary glands in 10 μl of PBS and 10^6 ^stationary phase *L. major *promastigotes in 50 μl of PBS inoculated subcutaneously in the right hind footpad. The footpad swelling at the site of inoculation was monitored at weekly intervals using a vernier calliper. The lesion size was defined as the increase in the footpad thickness after subtracting the size of the contralateral uninfected footpad. These experiments were repeated three times.

### Evaluation of parasite load

Parasite load was evaluated for all groups of mice. At the seventh week post-infection, three mice per each category (F2, F3, F4, F5, F6, F14, and their respective control groups) were used to determine the parasite load. For each mouse, parasite burden was assessed for the following tissues: footpad lesion, lymph nodes, and spleen. The number of viable parasites present at the site of infection (footpad, draining lymph nodes, and spleen) was quantified using the limiting dilution method [16]. Briefly, each pool of tissue was excised and homogenized in RPMI medium supplemented with 20% heat-inactivated foetal bovine serum, 100 U of penicillin per ml and 100 μg of streptomycin per ml. Each tissue homogenate was serially diluted in a 96-well Maxisorb plate (Nunc, Roskilde, Denmark). Samples, in quadruplicate, were incubated at 23°C. The wells containing motile promastigotes were identified under the microscope, and the number of viable parasites in each tissue was determined from the highest dilution at which promastigotes had grown after up to seven days of incubation. Results were expressed as the mean -log_10 _parasite titer.

### Statistical analysis

Using a linear mixed-effects model for longitudinal data, and while allowing for nested random effects (random intercept), and whereas the within-subject residual errors are permitted to be correlated (autoregressive of the first order, AR1) and/or have unequal variances [17], we tested for difference in trends (generation effect) as well as time-generation interaction, between curves illustrating the variation of the lesion size through time for each group of mice immunized and challenged differently as described above. In addition, for specific time point analysis (e.g., post-challenge starting at 3^rd ^week), Wilcoxon [18] and Student's t-test were used to determine median and mean differences in lesion size between groups. Maximum p-value is reported when difference is significant and minimum p-value when it is not. In addition Holm's correction for multiple testing, of the reported p-values was done when appropriate. The test data considered for the analysis consisted of subsets involving different generations combined sequentially starting from F2, and F3 data. The criteria when the model detect a generation effect is a p-value < 0.05, associated with generation effect. This will show that there is a new generation effect, i.e. a transition from neutral or lack of protection to protective effect. Considering the difference in lesion between groups for post-challenge starting from 3^rd ^week, the same linear mixed model was applied to the obtained difference, to confirm the finding of the above approach. Alternatively, to test for trend or level change i.e. a departure of the mean difference from stationarity, KPSS test was used [19]. To evaluate and test the correlation between parasite load and lesion size, Pearson's statistic was used. All the statistical analyses were performed with the following packages (Stats, Nlme, and Tseries) implemented in the R software [version 2.10.1] for statistical computing (http://www.r-project.org).

## Results

**Figure 1:**
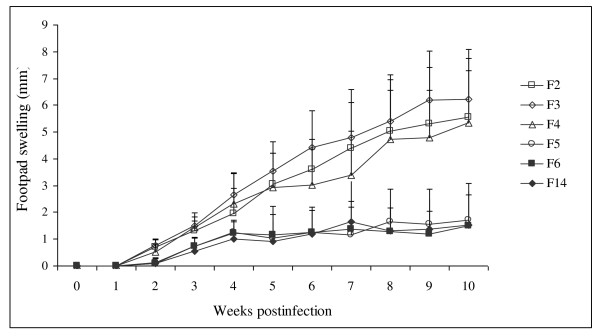
**Lesion progression in vaccinated BALB/c mice after challenge with 10^6 ^*L. major *metacyclic enriched promastigotes**. Each experiment is repeated three times. Results of the 3 experiments (10 mice per group) pooled together are expressed as increases in footpad thickness (in millimetres) and are means + S.D. F2: mice pre-immunized with SGH of female *P. papatasi *(F2) and challenged with *L. major *co-inoculated with the same type of SGH; F3: mice pre-immunized with SGH of female *P. papatasi *(F3) and challenged with *L. major *co-inoculated with the same type of SGH; F4: mice pre-immunized with SGH of female *P. papatasi *(F4) and challenged with *L. major *co-inoculated with the same type of SGH; F5: mice pre-immunized with SGH of female *P. papatasi *(F5) and challenged with *L. major *co-inoculated with the same type of SGH; F6: mice pre-immunized with SGH of female *P. papatasi *(F6) and challenged with *L. major *co-inoculated with the same type of SGH; F14: mice pre-immunized with SGH of female *P. papatasi *(F14) and challenged with *L. major *co-inoculated with the same type of SGH;

**Figure 2:**
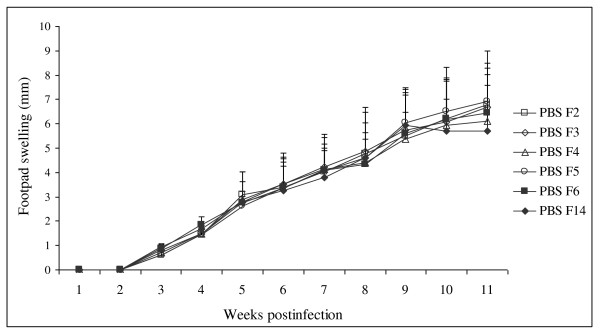
**Lesion progression in control BALB/c mice after challenge with 10^6 ^*L. major *metacyclic enriched promastigotes**. Each experiment is repeated three times. Results of the 3 experiments (10 mice per group) pooled together are expressed as increases in footpad thickness (in millimetres) and are means + S.D. PBS-F2 (control group): mice pre-immunized with PBS only and challenged with *L. major *co-inoculated with SGH of female *P. papatasi *(F2); PBS-F3 (control group): mice pre-immunized with PBS only and challenged with *L. major *co-inoculated with SGH of female *P. papatasi *(F3); PBS-F4 (control group): mice pre-immunized with PBS only and challenged with *L. major *co-inoculated with SGH of female *P. papatasi *(F4); PBS-F5 (control group): mice pre-immunized with PBS only and challenged with *L. major *co-inoculated with SGH of female *P. papatasi *(F5); PBS-F6 (control group): mice pre-immunized with PBS only and challenged with *L. major *co-inoculated with SGH of female *P. papatasi *(F6); PBS-F14 (control group): mice pre-immunized with PBS only and challenged with *L. major *co-inoculated with SGH of female *P. papatasi *(F14);

Footpad lesions of mice pre-immunized with SGH of F5, F6, and F14 female *P. papatasi *developed after challenge with *L. major *co-inoculated with each of the three types of SGH, but they were significantly smaller in size and grew more slowly than in the control groups (max p-values < 0.0001) (Figures 1, 2). In contrast, mice pre-immunized with SGH of F2, F3 female *P. papatasi *challenged with *L. major *co-inoculated with each of the two types of SGH developed lesions as rapidly and as large in size as the control groups (p = 0.67) (Figures 1, 2). Mice pre-immunized with SGH of F4 female *P. papatasi *challenged with *L. major *co-inoculated with the same type of SGH developed lesions less rapidly and less large in size as the control groups, and those pre-immunized with SGH of F2 and F3 female *P. papatasi*, but without significant differences (min p-values = 0.1). However, lesions size observed in the group of mice pre-immunized with SGH of F4 female *P. papatasi *are larger in size and grow more rapidly than the ones observed in the groups of mice pre-immunized with SGH of F5, F6, and F14 female *P. papatasi *(p-value < 0.0001) (Figures 1, 2). Lesions size differed significantly in mice pre-immunized with SGH of F2, F3 compared to lesions size observed in the groups of mice pre-immunized with SGH of F5, F6, and F14 female *P. papatasi *(max p-value < 0.0001).

**Figure 3:**
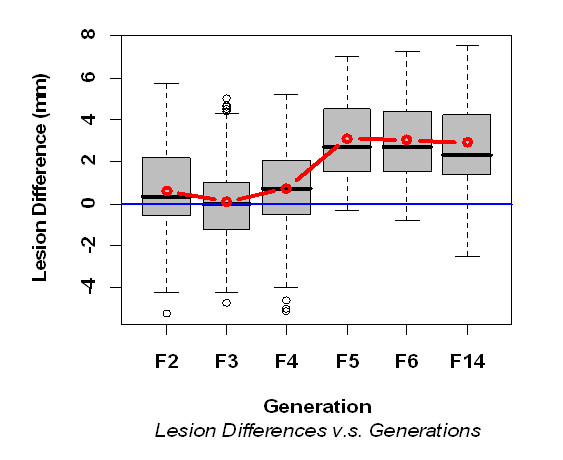
**Box-plot of the lesion difference between PBS pre-immunized group and challenged with Fi SGH and Fi pre-immunized group versus the generation Fi**.

Considering as test data, subsets involving different generations combined sequentially starting from F2, and F3 data, and the response variable as the difference in lesion size between group pre-immunized with SGH of Fi and its control group, a generation effect is detected at F4 (p-value = 0.019). This showed that there is a transition from lack of protection to protection. This protective effect phenomenon became clearer starting from the F5 (max p-values < 0.001) (Figure 3). Alternatively, the KPSS test for trend and level change indicated a level change, i.e. a departure of the mean difference from stationarity (p-value = 0.036) (Figure 3).

**Figure 4:**
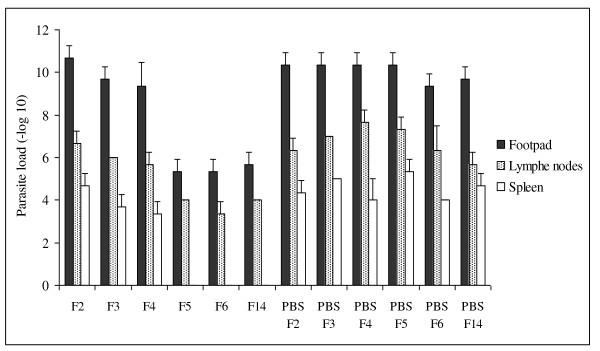
**Parasite loads**. At seven weeks post-infection, the number of viable parasites was determined from footpad, draining lymph nodes, and spleen of mice pre-immunized with SGH of F2, F3, F4, F5, F6, F14, and control groups. The parasite burdens in the infected tissues were assessed by limiting dilution, as described in Material and Methods. Presented values are the mean +S.D. of three mice per category.

The lesion sizes among different groups were proportionally correlated with parasite burdens in different organs i.e. footpad, lymph nodes, and spleen; (min r = 0.88, max p < 0.001) (Figure 4). A statistically significant difference (max adjusted-p < 0.001) in mean parasite load in the infected footpad of up to 4 log_10 _units was observed in mice pre-immunized with SGH of F5, F6, F14 compared to their control groups respectively, and to those pre-immunized with SGH of F2 and F3 female *P. papatasi*. A significant difference (max adjusted-p < 0.05) in mean parasite load in draining lymph nodes of up to 3 log_10 _units was observed in mice pre-immunized with SGH of F5, F6, F14 compared to their control groups respectively, and to those pre-immunized with SGH of F2 and F3 female *P. papatasi*. Similarly, a significant difference (max adjusted-p < 0.01) in mean parasite load in the spleen of up to 4 log_10 _units was observed in mice pre-immunized with SGH of F5, F6, F14 compared to their control groups, and to those pre-immunized with SGH of F2 and F3 female *P. papatasi*. The parasite loads in infected footpad, lymph nodes, and spleen were significantly lower in mice pre-immunized with SGH of F5, F6, and F14 than in the group of mice pre-immunized with SGH of F4 female *P. papatasi *(max adjusted p < 0.05). There is a significant difference in parasite load observed in mice pre-immunized with SGH of F4 compared to the groups of mice pre-immunized with SGH of F2 female *P. papatasi*. The difference in parasite load observed between mice pre-immunized with SGH of F4 and to those pre-immunized with SGH of F3 female *P. papatasi *is not statistically different for the footpad and lymph node but it is statistically significant for the spleen (p < 0.05). No significant difference in parasite load was observed between mice pre-immunized with SGH of F2 and F3 and the control groups (p > 0.05) for all tissues tested (Figure 4).

## Discussion

Our results demonstrate that pre-immunization with SGH of F2, and F3 generations of female *P. papatasi *does not confer protection against *L. major*. Similarly, pre-immunization with SGH of wild-caught or recently colonized *P. papatasi *(F1) do not confer protection against *L. major *infection [11]. The shift from lack of protection to protection occurs at the F4 generation and the protection effect of SGH is first observed at F5 and in following generations. This shift occurring at the F4 generation was within this particular study and the timing is likely to vary from case to case depending on the selection applied and ease/difficulty of adaptation of sand flies from the wild to a laboratory colony. As far as we are aware, this is the first report on the effect of colonization on the outcome of pre-immunization with SGH on *L. major *infection.

Previous studies showed that pre-immunization with SGH, salivary component, or pre-exposure to uninfected bites of long-term laboratory-colonized female *P. papatasi*, induced significant protection against *L. major *co-inoculated with the same type of SGH [8-10]. These studies were performed with long-term colonized *P. papatasi*. We showed that pre-immunization with SGH of recently colonized *P. papatasi *did not provide protection against *L. major *co-inoculated with the same type SGH compared to a significant protection obtained with SGH of long-term colonized one [11]. The protective effect against *L. major *following pre-exposure of mice twice to uninfected bites of long-term colonized *Phlebotomus dubosqi *shortly before experimental challenge provided protection against *L. major *[20]. However, there is a lack of protection when mice are pre-exposed for long-term to uninfected bites of *P. dubosqi *followed by free sand fly bites period prior to infection [20]. It was reported that pre-immunization of BALB/c with SGH of colonized *Lutzomyia intermedia *did not protect BALB/c mice against *Leishmania braziliensis *co-inoculated with the same type of SGH [21]. The use of SGH may represent a hurdle in the development of vaccines based on sand fly saliva and therefore, it is highly needed to identify and select individual salivary protein candidates instead of using SGH [21]. It is important to point out that these authors emphasized that colonization of *Lu. intermedia *is a challenging process [21]. Thus, the enhancement observed for *L. braziliensis *infection following pre-exposure of mice to *Lu. intermedia *saliva was potentially due to the sand fly generation used which was not indicated in their study.

As for ticks, it is well established that there are homologues and prologues in their salivary gland trasncriptomes that likely encode products to circumvent host immune responses that could neutralize their biological activity [22]. Natural selection may occur in natural field populations of sand flies that favor polymorphism of salivary gland proteins and subsequently induce antigenic variation to avoid effects of the host immune system [23,24]. This hypothesis is corroborated by our findings showing: 1) that pre-immunization with SGH of wild-caught or recently colonized (F1) *P. papatasi *do not confer protection against *L. major *compared to a significant protection obtained with long-term colonized ones, [11] and 2) pre-immunization with SGH of long-term colonized (F39) *P. papatasi *do not confer protection against *L. major *co-inoculated with SGH of wild-caught compared to a significant protection obtained when both pre-immunization and challenge were performed with SGH of long-term colonized *P. papatasi *[25]. It was also reported that colonized and wild-caught *Lutzomyia longipalpis *differ in the composition and the amount of salivary proteins and these differences may account for the lower effect observed on the modulation of experimental *Leishmania *infection by wild-caught SGH [26,27].

Despite that fact that no direct evidence is presented in this study for a loss of variability in salivary protein genes, we hypothesized that a loss of genetic variation as a result of colonization is potentially responsible for the protection observed in mice pre-immunized with long-term colonized *P. papatasi*. Conversely, the antigenic diversity of salivary gland proteins of recently colonized *P. papatasi *is likely the reason for lack of protection in mice pre-immunized with SGH of recently colonized flies.

SP-15 was shown to be protective against *L. major *[10]. It was hypothesized that the development of a vaccine based on SP-15 will not be affected by an inconsistent immune response due to genetic variation in natural populations of *P. papatasi *[28]. However, several studies emphasized that natural genetic variation in candidate salivary vaccines is an important issue in the potential efficacy of a vaccine [11,23-25].

Laboratory colonies of insects are often accepted as being representative of field populations from which they have been derived. However, this assumption may not always be valid, as colonies frequently incorporate only a fraction of the original population's genetic variability [29]. Long-term term colonization of *P. papatasi *induced a selection of refractory and susceptible lines to *L. major *[30,31]. Wild-caught *P. papatasi *exhibited the highest genetic variation in SP-15 compared to colonized flies of the same species [28]. Moreover, the analysis of genetic variation at 17 enzyme loci of one colonized and five field populations of *P. papatasi *showed that polymorphism of the examined loci observed in colonized and in field populations were 23.5% and 76.6%, respectively [32].

For the New World sand fly *Lu. longipalpis*, colonization led to reduced genetic variability in comparison to field samples, and to fixation of rare or previously undetected alleles [33]. Hence, colonization of sand flies may reduce genetic variability and may select for certain traits not present in field populations.

For *P. papatasi*, pre-immunization of mice to PpSP15 was shown to be protective against *L. major*, while immunization with another salivary gland protein PpSP44 from the same colony of *P. papatasi *induced disease enhancement [34]. In addition, pre-immunization with SGH of wild-caught or recently colonized *P. papatasi *does not protect against *L. major *infection contrasting with significant protection observed with long-term colonized flies [11]. Our previous studies revealed that pre-immunization with SGH of long-term colonized (F39) *P. papatasi *do not confer protection against *L. major *co-inoculated with SGH of wild-caught compared to a significant protection obtained when both pre-immunization and challenge were performed with SGH of long-term colonized *P. papatasi *[25].

In conclusions, we provide in this study further evidence that colonization has a direct impact on the outcome of *L. major *infection following pre-immunization with SGH of different generation of *P. papatasi*. Moreover, the change in the resulting effect was detected between the 4^th ^and 5^th ^generations following colonization. As indicated above, whether this is related to a qualitative or quantitative difference in the sand fly saliva remains to be determined.

## Competing interests

The authors declare that they have no competing interests.

## Authors' contributions

SBM carried out the experiments, BK, performed the statistical analysis, IC, SC, MD are maintaining the colony of sand fly and salivary gland dissections, DL provided *L. major *isolates used in this study, EZ designed the experiment and drafted the first version of the manuscript and finalized the manuscript. All authors read and approved the final version of the manuscript.
